# An observational study to understand burden and cost of care in adults diagnosed with refractory chronic cough (RCC) or unexplained chronic cough (UCC)

**DOI:** 10.1186/s12931-024-02881-4

**Published:** 2024-07-04

**Authors:** Jaclyn A. Smith, Norman Stein, Sylwia Migas, Sue Bokowski, Claire Williams, Patricia Baker, John New, Jonathan Schelfhout, Eileen Fonseca, Haya Langerman

**Affiliations:** 1 Division of Immunology, Immunity to Infection and Respiratory Medicine, University of Manchester, Manchester University NHS Foundation Trust, Manchester, UK; 2 Division of Infection, Immunity and Respiratory Medicine, University of Manchester, Education and Research Centre, Manchester University NHS Foundation Trust, Wythenshawe Hospital, Manchester, UK; 3 NorthWest Ehealth Ltd, Manchester Science Park, Manchester, UK; 4 Salford Royal NHS Foundation Trust, Salford, UK; 5 Merck & Co., Inc., Rahway, NJ USA; 6 MSD (UK) Limited, London, UK

**Keywords:** Chronic cough, Cost, Healthcare utilisation, Refractory chronic cough, Unexplained chronic cough

## Abstract

**Background:**

Refractory and unexplained chronic cough (RCC and UCC) necessitate frequent referral for specialist evaluations, but data on healthcare resource utilisation and costs are lacking.

**Methods:**

This observational study enrolled adults with RCC or UCC attending a specialist cough clinic and included a control cohort, both from North West England, matched 1:5 for age, gender and smoking history. Primary and secondary care data were obtained for the 5 years prior to and 2 years post initial clinic visit (index). The primary endpoint was the total 5-year healthcare cost to the UK NHS pre-RCC or UCC diagnosis compared to the control cohort.

**Results:**

Mean age at index for the 200 RCC or UCC consented patients was 62.2 ± 11.4 years; 71% were female, and 68% had never smoked. Mean duration of symptoms pre-diagnosis was 8.0 ± 9.4 years. Mean cough severity score was 63.7 ± 23.2 mm at index on a Visual Analog Scale, and Leicester Cough Questionnaire total score was 10.9 ± 4.1. GP data were available for 80 patients and mean total cost over the 5 years pre-diagnosis (index date) was 3.0-fold higher (95% CI 2.3, 3.9) than in the control cohort (*p* < 0.001). Most excess costs were related to visits and procedures carried out in secondary care. RCC- or UCC-associated costs decreased post-diagnosis, but remained higher than those of controls.

**Conclusion:**

Diagnosis of RCC or UCC requires significant health resource utilisation in the 5 years prior to a specialist clinic diagnosis. Resource utilisation was less after diagnosis, but remained higher than in a matched control cohort.

**Supplementary Information:**

The online version contains supplementary material available at 10.1186/s12931-024-02881-4.

## Introduction

Frequent coughing over a prolonged period without resolution can have significant physical, social and psychological consequences [1], including sleep disturbance, urinary stress incontinence, anxiety, depression, and interference with work/socializing [2–5]. Social isolation may also be a factor, particularly since the onset of the coronavirus disease 2019 pandemic [6].

In clinical practice it is important to distinguish between cough that is truly refractory or unexplained, and cough that can be explained and treated effectively. Refractory chronic cough (RCC) is a cough that persists despite investigations and guideline-based treatment of common underlying causes such as asthma, chronic obstructive pulmonary disease (COPD), airway hyper-responsiveness, eosinophilic bronchitis, rhinitis, angiotensin-converting enzyme inhibitor treatment, gastro-oesophageal reflux disease (GORD), and obstructive sleep apnoea [7–9]. In other patients, clinical assessment may fail to identify a cause after diagnosis and treatment by evidence-based guidelines, and patients are classified with unexplained chronic cough (UCC) [8, 9]. Limited prevalence data suggest that RCC might account for a third of chronic cough patients and UCC for about 10% [5, 10].

RCC and UCC can have a major economic impact on healthcare systems and society. However, data on the burden of RCC and UCC in terms of healthcare resource utilisation is lacking. The demonstration of both medical and economic value is important for clinicians, healthcare providers, payers, and patients as new therapeutic agents for these conditions approach late-stage clinical trials. This observational study was conducted to assess the burden of RCC and UCC to the healthcare system by analysing resource utilisation and treatment patterns associated with these conditions in the 5 years prior to and 2 years post first attendance at a specialist clinic.

## Methods

This was a single centre, observational, case-control study, conducted at the Manchester University NHS Foundation Trust (MFT) cough clinic, a secondary care setting treating patients from across North West England. Patients new to the clinic and diagnosed with RCC or UCC between September 2017 and June 2019 were identified from a review of the clinic’s medical records to confirm eligibility. Patient data (including demographics, history, investigations and previous treatment trials) were collected using a standard proforma which also recorded the presence or absence of complications of coughing such as cough-induced urinary incontinence. The diagnosis of RCC or UCC was made using a local algorithm based on British Thoracic Society Guidelines [11]. Participants were required to be ≥ 18 years of age and have data available on the severity and duration of RCC or UCC at the time of diagnosis (baseline).

Primary care data for the RCC and UCC cohort came from GP practice and secondary care data were obtained from the cough clinic proforma, the Hospital Episode Statistics (HES) database, and GP electronic medical records.

A control cohort was created by matching five control subjects from the Salford area of North West England to each RCC and UCC participant by year of birth, gender and smoking status. Controls were required to have at least 5 years of medical record data for the period preceding the date of diagnosis of their matched RCC or UCC case. The controls were otherwise selected at random. For the RCC and UCC cohort, the index date was the date of diagnosis. For the control group, the index date was the RCC or UCC diagnosis date of the case with whom they were matched. Controls were identified using the Salford Integrated Record (SIR). Further details on the procedure for identification of the cohorts are provided in Supplementary document 1.

A total of 200 patients from the cough clinic provided written, informed consent and were recruited to the full RCC and UCC cohort and matched with 1000 controls. GPs were asked to consent to extraction and transmission of primary care data; this was received for 80 of the 200 patients comprising the RCC and UCC cohort. Analyses requiring use of primary care data, including the primary endpoint, were therefore restricted to this subset and 400 matched controls. The remaining analyses used the full 200 RCC or UCC and 1000 control patient cohorts.

### Costing procedure

Total healthcare costs to the UK NHS associated with RCC or UCC were determined, including those arising from outpatient appointments (clinic conducted on hospital premises with consultant physician from appropriate specialty), day-case visits (not requiring use of a hospital bed overnight), primary care visits (GP and nurse consultations), and cough-associated prescription costs. Costs for non-elective and elective hospital admissions were excluded from this study, on the basis that RCC or UCC is not a condition normally requiring hospital admission as an inpatient. Appointments that were cancelled, or where the patient did not attend, were excluded. It was assumed that a patient could not have more than one visit to a particular specialty on a given day. Further details of the costing procedure are provided in Supplementary document 1.

### Cough severity measures

RCC and UCC severity and impact at the time of diagnosis were determined using the cough severity Visual Analog Scale (VAS) and Leicester Cough Questionnaire (LCQ). The cough severity VAS uses a 100-mm linear scale ranging from “no cough” (0 mm) to “worst cough” (100 mm) [2]. The LCQ is a 19-item cough-specific health-related quality-of-life questionnaire comprising three domains that assess the impact of cough on physical, psychological, and social functioning, with a recall period of the past 2 weeks. Each item is rated using a 7-point scale and the total score, calculated by summing the domain scores, ranges from 3 to 21 with a lower total score indicating greater impairment of health status due to cough [2].

### Study endpoints

The primary endpoint was the total 5-year healthcare cost pre-RCC or UCC diagnosis (defined as the cost of outpatient and day-case clinics, attendance at GP surgeries, and primary care drug costs) for the sub-cohort of patients for whom primary healthcare data was available in the 5 years before diagnosis compared to a matched control group.

A number of secondary endpoints were also evaluated including: secondary care costs (combined cost of outpatient visits and day-case admissions, 5 years pre-index date); number of outpatient and day-case visits by specialty (e.g. respiratory; ear, nose and throat; gastroenterology); correlation between VAS and LCQ scores at baseline and total cost over the 5-year pre-diagnosis period; and healthcare costs (both total and secondary care) for 2 years post-diagnosis, analysed as four consecutive 6-month intervals.

### Statistical analyses

The total 5-year healthcare cost pre-RCC or UCC diagnosis was calculated using a generalized linear model (GLM) with log link and underlying gamma distribution. As is typical of cost data, the distribution of data was right skewed with some participants accruing a very high cost. The GLM approach uses log transformation to normalize the distribution of notably skewed costs. The mean, treatment ratio (predicted cost for cases divided by the predicted cost for controls) and associated p-value and 95% confidence interval were determined. The standardized mean difference (SMD [12]) is provided as a measure of the size of the difference. As RCC and UCC cases were drawn from all over North West England whereas controls were restricted to the Salford area, a sensitivity analysis was performed using the Charlson Comorbidity Index (CCI) as a potential confounder to explore the generalisability of the results [13, 14].

Approval from the Health Research Authority (HRA), and South Central - Hampshire A Research Ethics Committee was sought and obtained prior to any study activities commencing. Patients or the public were not involved in the design, conduct, reporting or dissemination plans of our research. Written informed consent was obtained from all patients who participated in the study.

## Results

### Baseline demographic and clinical characteristics

Baseline demographic and clinical characteristics for the RCC and UCC as well as control cohort are illustrated in Table 1. Demographic and clinical characteristics for the sub-cohort of RCC and UCC patients with primary care data were very similar to the full cohort and well matched with controls (Table 1).

**Table 1:** Cohort demographics and baseline characteristics. Since primary care data are the main source of information about comorbidities, prevalence data for the full cohort are unavailable. COPD, chronic obstructive pulmonary disease

		Full cohort		Sub-cohort with primary care data	
		Cases *N* = 200	Controls *N* = 1000	Cases *n* = 80	Controls *n* = 400
Sex	Female (%)	142 (71.0%)	709 (70.9%)	58 (72.5%)	289 (72.3%)
	Male (%)	58 (29.1%)	291 (29.1%)	22 (27.5%)	111 (27.7%)
Age [years]	Mean ± SD	64.25 ± 11.42	64.25 ± 11.38	64.50 ± 11.06	64.50 ± 11.01
Age at index date [years]	Mean ± SD	62.16 ± 11.41	62.16 ± 11.37	62.34 ± 11.05	62.34 ± 11.00
Smoking Status	Current smoker	6 (3.0%)	20 (3.0%)	* (*%)	10 (2.5%)
	Ex-smoker	58 (29.0%)	291 (29.1%)	22 (27.5%)	109 (27.3%)
	Never smoked	136 (68.0%)	679 (67.9%)	56 (70.0%)	281 (70.3%)
Ex-smokers (smoke free years)	Mean ± SD	22.88 ± 13.21		20.94 ± 11.57	
Comorbidities present in the 5 years pre-index date					
Asthma				24 (30)	14 (3.5)
COPD				* (*)	7 (1.8)
Reflux / oesophagitis				8 (10.0)	9 (2.3)
Allergic rhinitis				7 (8.8)	7 (1.8)
Acute sinusitis				11 (13.8)	23 (5.8)
Chronic sinusitis				6 (7.5)	7 (1.8)

*Small number suppression. Non-zero, but ≤ 5

**Fig. 1:**
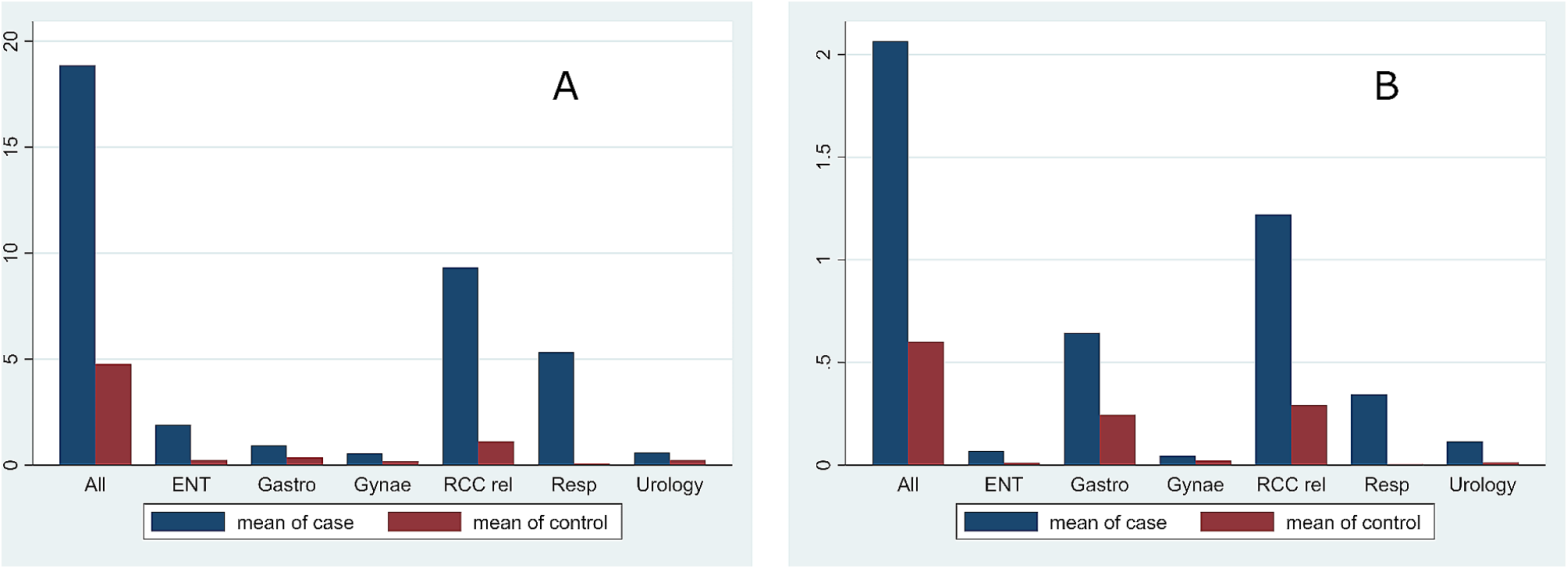
Mean number of (**A**) outpatient visits and (**B**) day-case visits in 5 years prior to diagnosis

Mean age at the time of RCC or UCC diagnosis was 62.2 (± 11.4) years (range 19 to 83 years) and the majority of patients were female (71%). The mean duration of troublesome cough symptoms before RCC or UCC diagnosis was 8.0 ± 9.4 years. Urinary incontinence affected 84 (59%) of the female study population. Cough severity VAS data were available for 190 (95%) patients at admission providing a mean (± SD) cough rating of 63.7 ± 23.2 mm. LCQ data relating to the time of admission to the specialist clinic were available for 128 (64%) patients. The mean (± SD) LCQ total score was 10.9 (4.1) indicating a moderate/severe impact of cough on quality of life, with mean scores of 4.1 (1.3), 3.4 (1.5) and 3.4 (1.6), respectively, for the Physical, Psychological and Social Domains (Fig. 1).

### Total cost (primary and secondary) in the 5 years pre-RCC or UCC diagnosis

The mean total cost over the 5 years prior to index (pre-diagnosis) for the RCC and UCC sub-cohort (*n* = 80) was £6010 (95% CI: £4557, £7463; median £4109) compared with £2032 (95% CI: £1812, £2251; median £1391) for the matched controls (*p* < 0.001). The GLM treatment ratio indicated cost was 2.96 times higher (95% CI 2.27, 3.85) in the RCC and UCC cohort than in the controls. The SMD for total cost was 0.912 indicating a large difference between groups.

The annual mean cost for controls increased slowly across the 5-year period, reflecting increasing cohort age. In contrast, mean annual costs for RCC and UCC cases increased sharply about 2 years prior to diagnosis (Table 2). Sensitivity analysis for total cost using CCI as an additional covariate showed little change from the original cost model.

**Table 2:** Mean annual total cost (for the subgroup with GP data) and secondary care cost in the RCC and UCC cohort for each of the 5 years prior to diagnosis. Year − 1 is the year immediately before diagnosis. RCC, refractory chronic cough. UCC, unexplained chronic cough

	RCC/UCC				Control			
	Total cost		Secondary care cost		Total cost		Secondary care cost	
	Mean	sd	Mean	sd	Mean	sd	Mean	sd
Year − 1	1577.20	1841.45	997.64	1300.27	462.32	672.05	237.01	588.21
Year − 2	1543.21	1849.23	976.93	1318.25	428.60	637.69	206.97	558.48
Year − 3	1013.17	1256.25	681.73	1037.48	417.47	625.69	195.27	503.43
Year − 4	942.52	1110.29	516.70	773.54	374.96	620.23	213.27	616.98
Year − 5	933.92	1248.29	552.83	967.65	348.10	594.42	187.87	515.50

### Secondary care costs

Secondary care costs were calculated for the full cohort of 200 patients and 1000 controls of whom 379 (4 RCC or UCC and 375 controls) had no secondary care costs. Mean secondary care costs over the 5 years prior to index were 3.58 times higher in the RCC and UCC cohort, with a mean (95% CI; median) cost of £3726 (£3112, £4340; £2458.5) for RCC or UCC cases and £1040 (£933, £1148; £309.5) for the controls.

### Healthcare visits and investigations

In the 5 years before an RCC or UCC diagnosis, there was a higher number of outpatient visits and day-case attendance for assessment by specialties managing chronic cough compared with controls. The mean number of RCC or UCC-related visits (Ear, Nose and Throat, Gastroenterology, Respiratory, Urology and Gynaecology combined) was 9.3 compared with 1.1 for controls; ratio 8.39 (95% CI 6.39, 11.02; *p* < 0.001) (Table 3). Chest X-ray and spirometry were the most frequently performed tests in the 5 years prior to diagnosis (Table 4). Outpatient and day-case visits to the individual specialties were also increased compared with controls (Fig. 1A and B).

**Table 3:** Mean number of outpatient visits in 5 years pre-diagnosis. RCC, refractory chronic cough. UCC, unexplained chronic cough

Visit type	RCC/UCC	Control	Ratio	95% CI		*p*
All	18.865	4.766	3.96	3.38	4.63	< 0.001
RCC related	9.320	1.110	8.39	7.08	9.93	< 0.001
ENT	1.895	0.239	7.93	6.35	9.90	< 0.001
Gastro	0.935	0.360	2.60	2.06	3.28	0.002
Respiratory	5.330	0.077	69.22	52.48	91.28	< 0.001
Urology	0.605	0.238	2.54	1.95	3.32	0.018
Gynaecology	0.555	0.197	2.82	2.13	3.71	0.008

**Table 4:** Number of tests (N), and frequencies of tests performed in the 5 years prior to diagnosis for RCC/UCC cases. CT, computer tomography. FeNO, fractional exhaled nitric oxide. RCC, refractory chronic cough. UCC, unexplained chronic cough. *Small number suppression. Numbers in the range 1 to 7 have been suppressed and are denoted by an asterisk

Test	*N*	0	1	1+	2	2+	3+
Chest X-ray	594	12	49		56		83
Spirometry (primary)	217	29	16		9		26
Spirometry (primary) scaled	543	73	40		22		65
Spirometry (secondary)	174	91	71		24		14
High resolution chest CT scan	127	83	107		10		0
Full lung function	91	126	61			13	
Bronchial challenge	30	170	30		0		0
FeNO	*						
Bronchoscopy	53	149		51			
Laryngoscopy	93	146	32		13		9
Nasendoscopy	92	138	44			18	
Gastroscopy	68	147	44			9	
24 h pH monitoring	11	192		8			

Similarly, the mean number of visits to the GP practice (GP and nurse consultations) in the 5 years prior to RCC or UCC diagnosis was higher among the RCC and UCC cohort compared with controls (51.8 versus 30.2 in the 5-year period, *p* < 0.001). This equates to an annual mean number of visits of 10.4 for RCC and UCC cases versus 6.0 for controls.

### Healthcare costs pre- and post-RCC or UCC diagnosis

The total cost (in the RCC and UCC sub-cohort) in the first 6 months post-diagnosis was significantly higher than in the 6 months immediately before diagnosis (Table 5). Most of these post-diagnostic costs related to laryngoscopy procedures. There were 75 laryngoscopies on 67 patients (8 had two each; the remainder had one each). The cost of a laryngoscopy was £141. This was the cost in the National Tariff for 2018-19.

**Table 5:** Total healthcare costs (£) and secondary care costs (£) in 6-month intervals pre- and post-diagnosis of refractory chronic cough or unexplained chronic cough (n is the number of patients included in each interval)

Interval (months)	Total healthcare costs (£), median [IQR]			
	n	Pre-Diagnosis	Post-Diagnosis	p
0–6	80	439 [243, 949]	855 [484, 1463]	< 0.001
6–12	80	555 [322, 872]	478 [247, 998]	0.335
12–18	79	450 [255, 846]	406 [178, 773]	0.068
18–24	55	412 [165, 871]	360 [154, 1039]	0.821

Interval (months)	Secondary care costs (£), median [IQR]			
	n	Pre-Diagnosis	Post-Diagnosis	p
0–6	200	209 [94, 539]	633 [302, 1329]	< 0.001
6–12	200	322 [ 143, 614]	282 [94, 718]	0.230
12–18	192	280 [94, 628]	225 [79, 574]	0.115
18–24	131	207 [0, 553]	135 [0, 548]	0.196

The costs in the subsequent 6-month periods up to 2 years post-diagnosis were lower than in the corresponding periods pre-diagnosis, although the comparison did not reach statistical significance. A similar pattern was observed when secondary care costs (total RCC and UCC cohort) were compared between the post- and pre-diagnosis periods (Table 5).

### Correlation between VAS and LCQ scores at baseline and cost

**Fig. 2:**
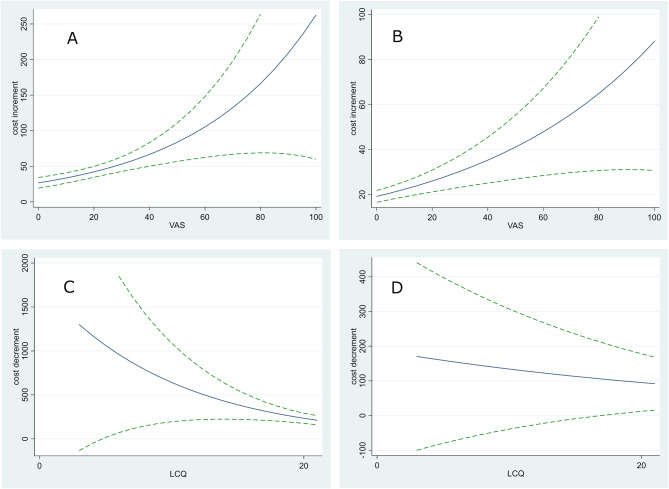
Mean increment in (**A**) total cost resulting from an increase in Visual Analogue Scale (VAS) score of 1 mm as a function of VAS score (blue line); (**B**) secondary care cost resulting from an increase in VAS score of 1 mm as a function of VAS score (blue line); (**C**) mean decrement in total cost resulting from an increase in Leicester Cough Questionnaire (LCQ) score of 1 as a function of LCQ score (blue line); (**D**) mean decrement in secondary care cost resulting from an increase in LCQ score of 1 as a function of LCQ score (blue line). The effect of increasing LCQ score on secondary care cost was not statistically significant. The broken green lines are 95% confidence intervals

The GLM model indicated there was a positive correlation between VAS score at baseline and total cost as well as secondary care cost (coefficients 0.0228 [95% CI 0.0132, 0.0325] and 0.0152 [95% CI 0.0089, 0.0216], respectively). The model predicted that, on average, an increase of 1 mm in the VAS score between patients was associated with a £133 increase in total cost and £55 increase in secondary care cost. The total cost and secondary care cost increments associated with unit increases in VAS score as a function of VAS score are shown in Fig. 2A and B.

There was also a correlation between LCQ score at baseline and total cost (coefficient − 0.1009, *p* = 0.001). Mean total cost was found to decrease by £635 when the LCQ score increased by 1 point between patients (with higher scores indicating less burden of cough) (Fig. 2C).

### Prescriptions of interest in the 5 years prior to diagnosis

**Fig. 3:**
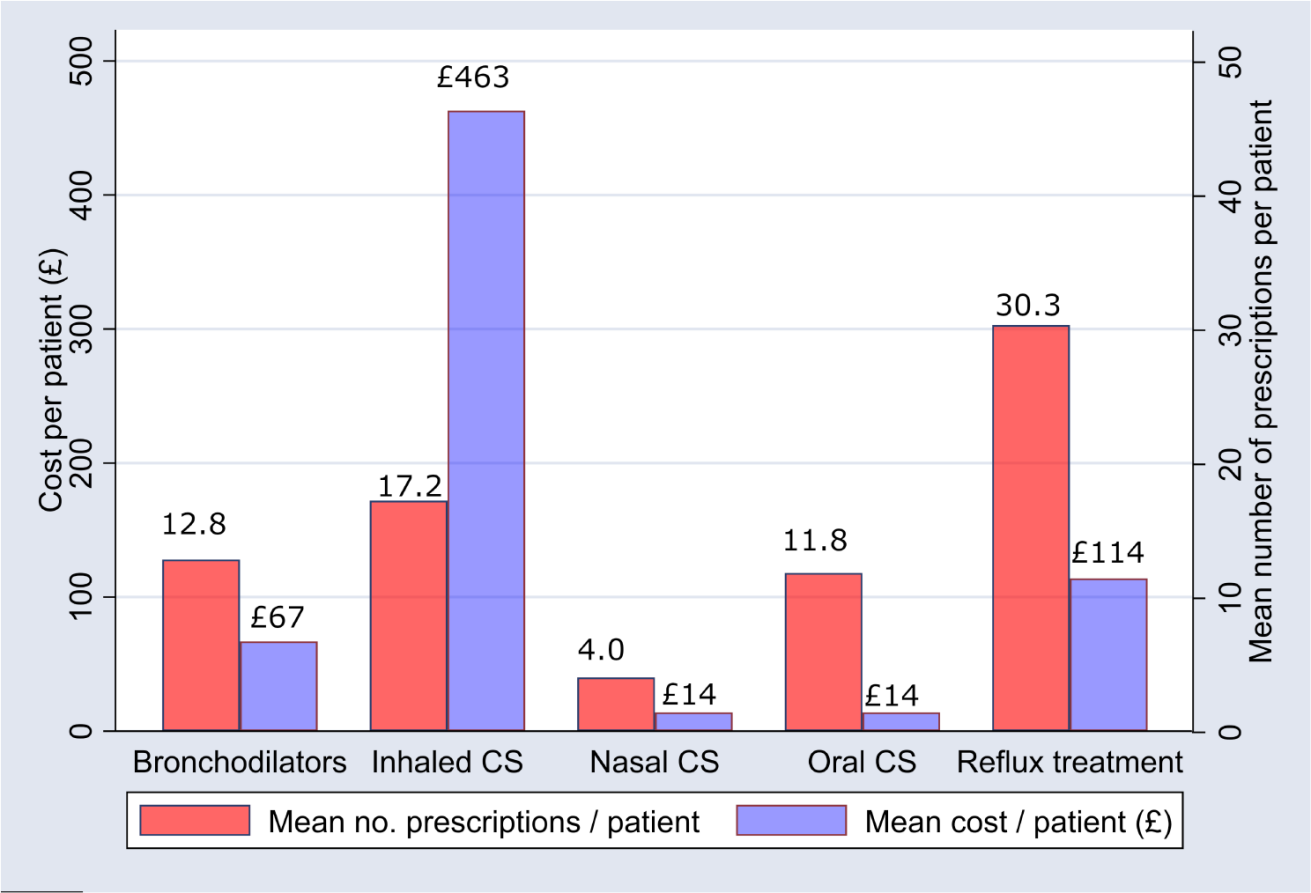
Mean number of prescriptions issued per patient and mean prescription cost (£) per patient in the 5 years prior to a cough clinic refractory chronic cough (RCC) or unexplained chronic cough (UCC) diagnosis. CS = corticosteroid

In the 5 years prior to RCC or UCC diagnosis, the total cost of prescriptions for medications of interest was £19,938, a mean cost of £100.2/patient. The most commonly prescribed agents were antisecretory drugs and mucosal protectants (e.g.: histamine H₂-receptor antagonists and proton pump inhibitors) with a mean of 30.3 prescriptions/patient at a mean cost of £114/patient (Fig. 3). Other frequently prescribed treatments were: inhaled corticosteroids (17.2 prescriptions/patient at a mean cost of £463), oral and nasal corticosteroids were also frequently prescribed; and bronchodilators (12.8 prescriptions/patient at a mean cost of £67/patient).

## Discussion

Data from this UK observational study show for the first time that patients’ total healthcare costs (primary and secondary care) in the 5 years leading up to a diagnosis of RCC or UCC were almost threefold higher than in a control group matched for age, gender and smoking status. These increased costs reflected greater numbers of primary care, outpatient and day-case visits as well as more prescriptions in the RCC and UCC cohort. Although RCC and UCC cases attended primary care more frequently than controls, most excess costs were related to secondary care visits and procedures where costs were 3.6-fold higher in the RCC and UCC cohort. Of note higher costs were associated with more severe cough severity and impact on quality of life.

A comparison of healthcare costs in 6-month intervals pre- and post-RCC or UCC diagnosis revealed that the total cost in the first 6 months post-diagnosis was significantly higher than in the 6 months immediately before diagnosis. The initial rise in costs immediately post-diagnosis reflects the costs of investigations conducted by the cough clinic, and a similar pattern was observed when secondary care costs were compared between the post- and pre-diagnosis periods. Most of these post-diagnostic costs related to laryngoscopy procedures carried out, not for diagnostic purposes, but as part of the evaluation for suitability for the cough suppression techniques taught by our speech and language therapists. Low total healthcare costs for the last 6-month period pre-diagnosis may reflect the fact that other treatment options have become exhausted immediately before cough clinic referral.

Just under three quarters (72.5%) of participants in this survey had persistent cough despite being diagnosed and treated for common chronic cough associated comorbidities, i.e. RCC. This was reflected in the medications prescribed which included inhaled, oral and nasal corticosteroids, bronchodilators, H₂-receptor antagonists and proton pump inhibitors, and antihistamines. The RCC and UCC cohort also presented with long-lasting (mean duration 8 years), severe (mean VAS 60.5 mm), and burdensome cough, as evidenced by low LCQ scores, indicating greater impairment of health status due to cough.

Identifying RCC and UCC cases can be challenging and few studies have documented the burden and cost of care in patients with chronic cough, and none included a control group. One study, which attempted to identify patients from primary care records, categorized patients as having possible or probable chronic cough and reported much higher healthcare costs (£3663 in a 12-month period) compared to our values for confirmed RCC and UCC [15]. These costs were mainly explained by inpatient admissions, more typical of patients with exacerbations of chronic respiratory diseases such as COPD and asthma, and not typical of RCC and UCC patients seen in specialist cough clinics.

Another primary care study of healthcare costs, which excluded prescription costs and inpatient care [16], consequently reported lower annual costs of £288 to £513 prior to the chronic cough and £469 to £718 in the 12 months post-diagnosis. Costs were greatest in those with reflux disease and least in those without identified co-morbid conditions.

Our findings are most consistent with data from a UK study of patients attending a specialist cough clinic, most of whom were diagnosed with RCC or UCC [17]. Healthcare costs of £1663 were reported in the 12 months following clinic assessment. This study lacked a control group, and whether the costs were pre- or post-diagnosis of RCC or UCC was unclear. Nonetheless, as in the current study, diagnostic investigations were the largest contributor to cost (63%), and cough severity and worse cough-related health status were associated with a significant increase in costs.

### Strengths and limitations

This study has some limitations. Primary care data were available for only 80 patients, as opposed to the 200 in the full cohort. It may have been possible to draw stronger conclusions regarding comparison of costs pre- and post-diagnosis had more data been available. The control and RCC and UCC cohorts were not from the same geographical area and had differing health status, which could have influenced healthcare costs. However, sensitivity analysis using CCI as an additional covariate in the cost model suggested any resulting bias was small.

A strength of this study was that the burden of cough was quantified in a well-defined group of patients with RCC or UCC by comparison with a control population matched for age, gender and smoking history. The Quality and Outcomes in Primary Healthcare (QOPH) clinical indicators demonstrate that chronic disease prevalence in North West England is comparable to the UK population overall, and ONS statistics indicate that the breakdown of the North West England population by ethnicity is similar to that of England and Wales as a whole. Patients were predominantly middle-aged women in keeping with findings from other UK specialist cough clinics [18]. The findings from this study are therefore likely to be generalisable to the whole of the UK and can be used by healthcare authorities nationwide.

## Conclusion

This study strengthens the limited information available on the excess healthcare resource utilisation and costs associated with chronic cough, providing specific information on those with RCC and UCC who are the most challenging to manage as licensed therapies are lacking. Before diagnosis in a cough clinic, patients suffer from RCC and UCC for many years, undergo multiple investigations and receive multiple prescriptions at significant cost. Healthcare resource utilisation reduces within 6 months of a formal diagnosis but remains at a higher level than in the general population highlighting the need for therapies able to target the underlying RCC and UCC disease mechanisms.

### Electronic supplementary material

Below is the link to the electronic supplementary material.

Supplementary Material 1

## Data Availability

The data are not publicly available due to privacy or ethical restrictions.
