# Supplementary material for: An observational study to understand burden and cost of care in adults diagnosed with refractory chronic cough (RCC) or unexplained chronic cough (UCC)
Source: Respir Res. 2024 Jul 4;25:265. doi: 10.1186/s12931-024-02881-4 (PMC11225373; doi:10.1186/s12931-024-02881-4)
Supplement: Supplementary file 1 — Supplementary Material 1 [file 12931_2024_2881_MOESM1_ESM.docx]

**Supplementary document 1**

**Procedure for identification of the refractory chronic cough (RCC) and unexplained chronic cough (UCC) cohort**

Primary care data for the RCC and UCC cohort came from GP practice and secondary care data were obtained from the cough clinic electronic case report form, the Hospital Episode Statistics (HES) database, and GP electronic medical records. The RCC and UCC consented cohort were identified within GP systems and within the HES dataset using NHS number.

**Procedure for identification of the control cohort**

A control cohort was created by matching five control subjects from the Salford area of North West England to each RCC and UCC participant by year of birth, gender and smoking status. Controls were required to have at least 5 years of data for the period preceding the date of diagnosis of their matched RCC or UCC case. For the RCC and UCC cohort, the index date was the date of diagnosis. For the control group, the index date was the RCC or UCC diagnosis date of the case with whom they were matched.

Controls were identified using the Salford Integrated Record (SIR). The SIR research database is a single, comprehensive repository for healthcare data populated by 45 GP surgeries and 1 large university teaching hospital. Access to the SIR database for research purposes is via a dedicated governance board. SIR control data were anonymised and were not linked to any other data sets.

**Costing procedure**

Each outpatient visit is associated with a Health Resource Group (HRG) code, which can be mapped into a cost in pounds using the National Tariff for the financial year in which the visit took place. Day-cases were also costed using HRG codes in the appropriate version of the National Tariff. All consultant-led outpatient clinics and day-case vists were included in the costing, regardless of specialty.

The cost of a consultation with a GP was obtained from the Personal Social Services Research Unit (PSSRU) report for 2018-2019 [Curtis and Burns, 2019], which assumes an average consultation length of 9.22 minutes. GP costs were obtained by multiplying the number of visits by a representative average cost per visit. The cost of a nurse consultation in a GP surgery was obtained from the same source and assumed that the consultation lasted 10 minutes on average.

Drug costs were derived from the National Health Service Business Services Authority (NHSBSA) dictionary of medicines and devices (dm+d) Version 12.0.0 through NHS Digital’s Technology Reference Update Distribution (TRUD). Where a drug had been discontinued, the cost prior to discontinuation was used.

Primary care costs 5 years pre-index date consisted of cost of visits to the GP surgery plus primary care prescribing of relevant drugs and were calculated for the sub-cohort of 80 patients with primary care data. Secondary care costs 5 years pre-index date were calculated for the full cohort of 200 RCC and UCC patients and 1000 controls. As the number of patients and controls with zero secondary care costs was 379 and exceeded 5% of the cohort size of 1200, a two-part model was used to fit secondary care cost, consisting of a logit model to predict whether a patient had zero cost, followed by a GLM to model the costs of those patients with non-zero cost. The number of outpatient and day-case visits by specialty were calculated using a GLM assuming an underlying negative binomial distribution (a probability distribution used with discrete random variables). The mean number of visits, treatment ratio and associated p-value and 95% confidence interval were determined. A GLM with log link and gamma distribution was also used to examine whether there was a correlation between VAS and LCQ scores at baseline and total cost over the 5-year pre-period. Healthcare costs for the RCC and UCC cases with both primary and secondary care data, and the full RCC and UCC cohort with secondary care data, were calculated for four consecutive 6-month intervals following diagnosis. These costs were then compared with those in the corresponding 6-month interval pre-diagnosis using a paired t-test, with costs grouped in the following 6-month increments: 0-6, 6-12, 12-18, 18-24.

Curtis LA, Burns A. (2019) Unit Costs of Health and Social Care 2019. PSSRU, Kent, UK, 176 pp. ISBN 978-1-911353-10-2.
